# Gravity-Based Precise Cell Manipulation System Enhanced by In-Phase Mechanism

**DOI:** 10.3390/mi7070116

**Published:** 2016-07-09

**Authors:** Koji Mizoue, Manh Hao Phan, Chia-Hung Dylan Tsai, Makoto Kaneko, Junsu Kang, Wan Kyun Chung

**Affiliations:** 1 Department of Mechanical Engineering, Osaka University, Suita 565-0871, Japan; mizoue@hh.mech.eng.osaka-u.ac.jp (K.M.); haopm@hh.mech.eng.osaka-u.ac.jp (M.H.P.); mk@mech.eng.osaka-u.ac.jp (M.K.); 2 Department of Mechanical Engineering, Pohang 790-784, Korea; junsu_kang@postech.ac.kr (J.K.); wkchung@postech.ac.kr (W.K.C.)

**Keywords:** cell manipulation, pressure control, microfluidics, cell deformability

## Abstract

This paper proposes a gravity-based system capable of generating high-resolution pressure for precise cell manipulation or evaluation in a microfluidic channel. While the pressure resolution of conventional pumps for microfluidic applications is usually about hundreds of pascals as the resolution of their feedback sensors, precise cell manipulation at the pascal level cannot be done. The proposed system successfully achieves a resolution of 100 millipascals using water head pressure with an in-phase noise cancelation mechanism. The in-phase mechanism aims to suppress the noises from ambient vibrations to the system. The proposed pressure system is tested with a microfluidic platform for pressure validation. The experimental results show that the in-phase mechanism effectively reduces the pressure turbulence, and the pressure-driven cell movement matches the theoretical simulations. Preliminary experiments on deformability evaluation with red blood cells under incremental pressures of one pascal are successfully performed. Different deformation patterns are observed from cell to cell under precise pressure control.

## 1. Introduction

Cell deformability has been used as an index for the diagnosis of diseases and patients’ conditions in medical research for decades [1]. The microfluidic system has recently become a popular platform for single-cell assessments because of high-throughput and other advantages [2]. Precise control of the microfluidic flow is often needed when it comes to manipulating cells for deformability evaluation. For example, Sakuma et al. developed an on-chip cell manipulation system which can manipulate cells with a resolution as accurate as 240 nm [3]. Monzawa et al. further improved the manipulation speed up to 130 Hz as to move cells along a sinusoidal trajectory in a microfluidic channel [4]. Although these systems have been successfully applied for the evaluation of cell deformability by observing cell deformation during the manipulation, such as in the cell fatigue test [5], only position-based control is not sufficient for detailed evaluation due to a lack of the information of the applied force on the cells. For improving the evaluation of cell deformability, it is important to have a precise pressure control system, so that the pressure applied to the cells in a microchannel can be directly controlled. This work is motivated by this background.

**Figure 1:**
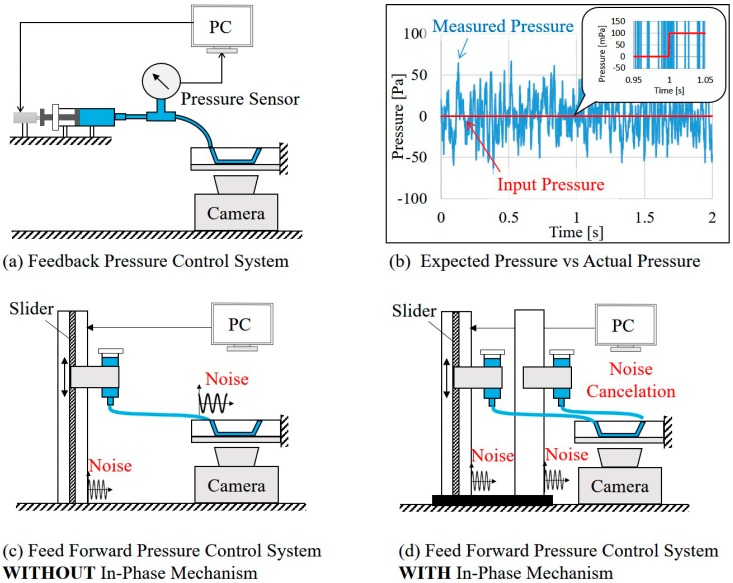
The conventional pressure system and the proposed system. (**a**) Feedback-controlled pressure system; (**b**) The control resolution is limited by sensor resolution. The blue signal is an example of measured pressure by a commercial pressure sensor; (**c**) Conventional gravity-driven pressure system; (**d**) The proposed system with in-phase noise cancelation.

Figure 1 shows the difference between a conventional system and the proposed system. Figure 1a illustrates a conventional system where the pressure at the channel inlet is controlled by a feedback controller with a pressure sensor. If the pressure is lower than the target value, the pump adds additional pressure, and vice versa. While a 100 mPa increase of pressure is intended to be applied to the channel inlet at the time of *t* = 1 s, as shown in Figure 1b, the system cannot effectively adjust the pressure because of the limit of the pressure resolution. The blue signal in Figure 1b is an actual example of measured pressure by a commercial pressure sensor with a mean low-pass filter. The resolution of the pressure is around 50 Pa, and as a result, 100 mPa manipulation is not possible.

Figure 1c illustrates a gravity-based pressure control system where the pressure is controlled by the height of the water head. The system is supposed to generate stable pressure outputs, and 100 mPa pressure can be achieved by simply adjusting the water head with a displacement of about 10 μm. However, the system is not practically adequate because ambient noise, such as stage the vibrations illustrated in Figure 1c, create pressure turbulence and compromises the expected high resolution. To cope with this issue, we introduce an in-phase noise cancelation mechanism where both the inlet and outlet are fixed on the same stage, as shown in Figure 1d. In this way, ambient vibrations to the system will affect both the water heads at the inlet and outlet at the same time. Since the pressure for driving the flow is only determined on the relative heights of the water heads, the problem with ambient noise is expected to be significantly reduced.

Experiments based on the proposed idea in Figure 1d were conducted. The water head of the inlet reservoir is controlled by a linear slider with a resolution of 10 μm while the water head of the outlet reservoir is fixed on the same stage. The pressure difference between the inlet and outlet of a microfluidic channel is experimentally determined according to the measured fluid flow in the microchannel. The system is also applied to the cell deformability test with pressure increments of 1 Pa, which is difficult to do using a conventional pressure control system. Human red blood cells (RBC) were tested, and cell behavior under a few pascals was observed experimentally.

The rest of the paper is structured as follows: After a brief review on the related works of cell evaluation, cell manipulation and conventional gravity-based microfluidic systems in Section 2, the working principle of the proposed precise pressure manipulation system and in-phase noise cancelation mechanism will be introduced in detail in Section 3. Experimental results including the effectiveness of the noise cancelation, the pressure-based cell manipulation and the evaluation of cell deformation under small pressure increments are presented in Section 4. The experimental results are discussed in Section 5. Finally, the paper is summarized with concluding remarks in Section 6.

## 2. Related Works

There are various approaches for single-cell evaluation and cell manipulation. For example, Sakuma et al. determined the RBC fatigue state by continuously pushing cells through a narrow channel using a high-speed syringe pump and a high-speed vision system [5]. Tan et al. measured the mechanical characteristics of RBCs under different osmotic pressure with optical tweezers [6]. Avci et al. achieved cell manipulation by dynamic release with chopstick-like microgrippers [7]. Tanyeri et al. developed a microfluidic Wheatstone bridge for rapid sample analysis [8]. Although these approaches demonstrate solid results in system functionalities, they require either costly experimental setup or great effort in system tuning and adjustments. On the other hand, gravity-based pressure/flow control for microfluidics has the great advantages of low cost, easy control and stability for the microfluidic system, and it has also been employed in the applications of cell evaluation. For example, Zhang et al. manipulated droplets by hanging reservoirs on a turn table [9,10]. Kang et al. controlled a micro-object by simple rotary arms [11]. Yamada et al. used stationary reservoirs as a constant pressure source for cell counting [12]. There are also works generating pressure with the water head difference using tilted microfluidic chips [13,14].

This paper successfully achieves the high-resolution control of pressure using a gravity-based system aimed for microfluidic applications, particularly in cell deformability testing. In addition, the in-phase noise cancelation mechanism is implemented in the system and experimentally evaluated.

## 3. Precise Pressure Control System with In-Phase Noise Cancelation 

### 3.1. The Gravity-Based Pressure Control

**Figure 2:**
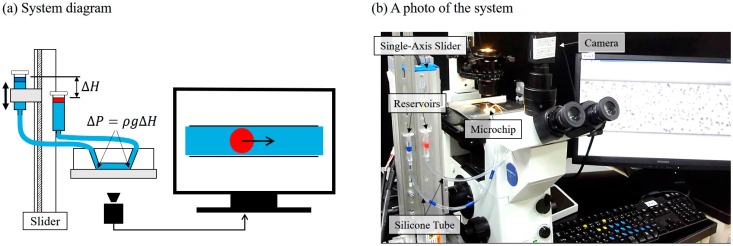
The overview of the experimental system. (**a**) Microfluidic flow is monitored through a camera while the slider is controlled by the signals from the computer; (**b**) A photo of the whole setup.

Figure 2a,b are a schematic diagram and a photo of the proposed system, respectively. The microfluidic flow is driven by the pressure difference between the two reservoirs as illustrated in Figure 2a. The pressure difference can be expressed by Pascal’s expression:

(1) ΔP=ρgΔH

where ΔP, ρ, g and ΔH are the pressure difference, the fluid density, the gravitational acceleration and the height difference between the water levels of the two reservoirs, respectively. Since the cross-sectional areas between the reservoirs ($2.83\;\times \;10^{7}\;{\text{μm}}^{2}$) and the microchannel ($3.00\times 10\;{\text{μm}}^{2}$) are about a million times different, the normal fluid flow in the microchannel is considered to not significantly affect the water head ΔH. For example, the flow rate of $600{\;\text{μm}}^{3}/s$, which is equivalent to about a 20 μm/s flowing speed of a suspended cell, would cause the change of ΔH at the speed of −0.02 nm/s. The change is less than 1% after 24 h continuous flow considering the ΔH is initially around 200 μm. 

The whole system setup shown in Figure 2b includes a polydimethylsiloxane (PDMS) chip where a microfluidic channel is fabricated, a microscope (IX71, Olympus Co., Tokyo, Japan), two syringe reservoirs and a single-axis slider (RSH205, Misumi Co., Tokyo, Japan). The precision of the slider is 10 µm, which gives a corresponding pressure of 100 mPa (assuming $\text{ρ}=10^{3}\;\text{Kg}\cdot {\text{m}}^{-3}$, $g=10\;\text{m}\cdot {\text{s}}^{-2},\;\text{Δ}H=10^{-5\;}m$). Both the inlet and outlet of the microfluidic channel are connected to the reservoirs fixed on the slider using silicone tubes, and the water head ΔH can be controlled by moving the inlet reservoir up or down. The slider body is made of aluminum and steel, and is considered a rigid body. In other words, it is assumed that there is no bending or any kind of deformation on the slider which may affect ΔH. The length and the outer and inner diameters of the tubes are 300, 6 and 4 mm, respectively. The placement of both the inlet and outlet reservoirs on the same stage, the slider, is the so-called in-phase noise cancelation mechanism which will be explained in the following section. 

### 3.2. The In-Phase Noise Cancelation

**Figure 3:**
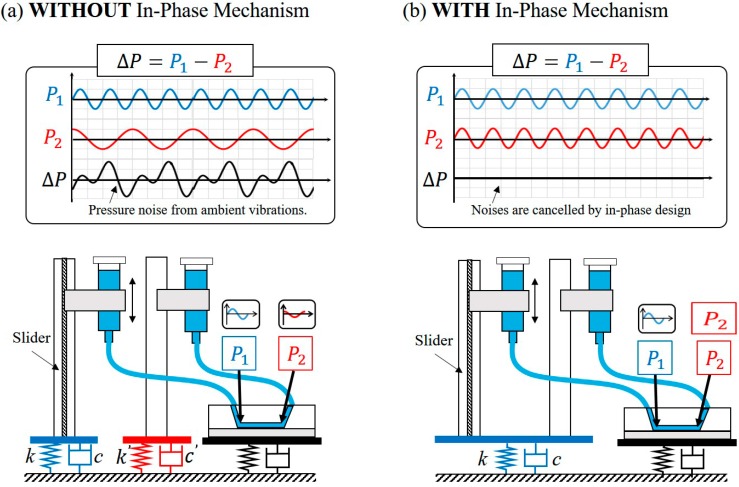
The idea of in-phase noise cancelation: (**a**) Without in-phase noise cancelation; (**b**) With in-phase noise cancelation.

Figure 3a,b illustrate examples without and with the in-phase noise cancelation, respectively. The spring-damper couples represent the mechanism of vibration on the stages where the reservoirs and microchannel are located. In Figure 3a, two reservoirs are placed on two different stages and, thus, different vibrations on them may happen even from the same vibration source. The pressure at the inlet and outlet of the microchannel, shown as $P_{1}$ and $P_{2}$ in Figure 3a, may change at different phases and magnitudes. As a result, the actual pressure driving the microfluidic flow would contain the vibration noises from both reservoirs as the resultant ΔP in Figure 3a. The in-phase noise cancelation is shown in Figure 3b where both reservoirs are placed on the same stage. No matter how the stage is vibrated by external noises, the heights of two reservoirs would change at the same phase and magnitude. Therefore, the pressure difference ΔP in Figure 3b is expected to be constant since the noises on the two reservoirs cancelled each other, and the constant height difference between the two reservoirs is maintained. We would like to note that since 100% in-phase cancelation is not possible in a practical situation, the experimental validation for such a mechanism is necessary for proving the effectiveness of such an in-phase noise cancelation.

## 4. Experiments

The experiments were performed using the system shown in Figure 2b. Both microbeads and RBCs are used in the experiments. The system is firstly validated by microbeads for the effectiveness of the in-phase noise cancelation. RBCs are later employed for determining the pressure-velocity relation as well as the deformability evaluation. The blood donor for RBCs has read and agreed to the consent of the experiment. The blood samples are taken by a commercial lancet 10 min before the experiment. The blood is diluted by standard saline (NaCl = 0.9%) at the ratio of 100:1 for single-cell evaluations. The zero point of the pressure (ΔP=0 and ΔH=0) is determined when the velocity of a suspended object is measured as zero in the microchannel. The motion of objects is recorded by a digital camera, and the recorded videos are analyzed using the Image-Processing Toolbox in Matlab.

### 4.1. Ambient Noises and the Effectiveness of Noise Cancelation

**Figure 4:**
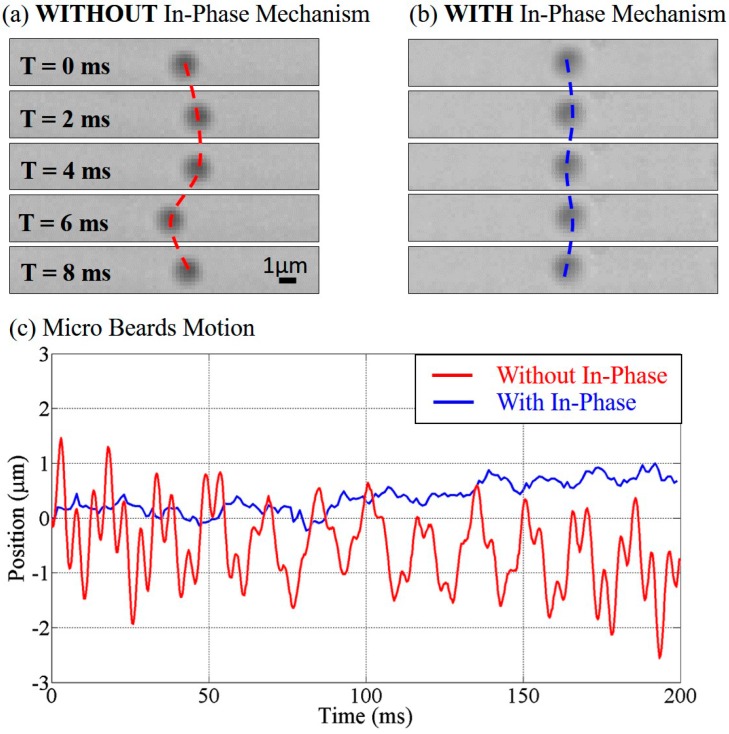
The motion of a microbead. (**a**) A microbead motion in the system without in-phase noise cancelation; (**b**) A microbead motion in the system with in-phase noise cancelation; (**c**) The tracked microbeads positions with respect to time.

Figure 4a,b show continuous snapshots of microbeads in the system without and with the proposed in-phase noise cancelation, respectively. No artificial noise was intentionally introduced in the experiments. The dashed line represents the tracked beads’ trajectories in this example, and clear zig-zag motions were observed in the system without the in-phase mechanism as shown in Figure 4a. Figure 4c shows the analyzed results from object tracking using image processing on recorded frames. In spite of drifting displacement, an average amplitude of 2 µm vibration has been found in the system without the in-phase mechanism while the vibration of the microbead in the system with the in-phase mechanism is less than 1 µm. From Figure 4, we can directly observe that a significant part of the beads’ vibration can be effectively removed with the proposed in-phase noise cancelation. 

**Figure 5:**
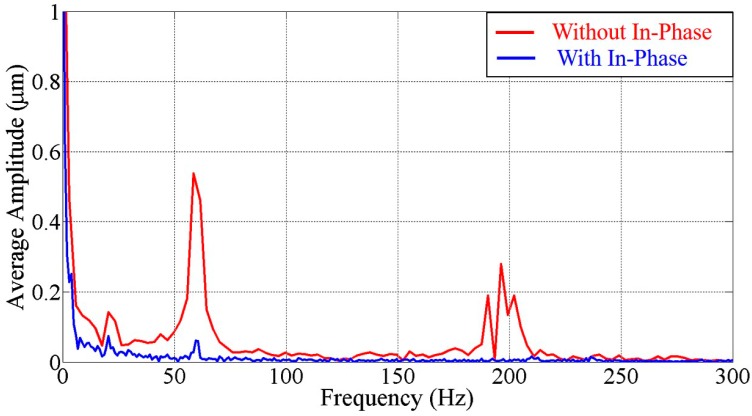
The frequency spectrum without and with in-phase noise cancelation.

Figure 5 shows the frequency spectrum derived from Figure 4 by fast Fourier transform. The red and blue lines represent the spectrum without and with in-phase noise cancelation, respectively. Three obvious peaks are found at the frequencies around 20, 60 and 180 Hz. Considering the experimental environment, they are suspected due to the vibrations from the microscope fan, the power supply for the slider, and the pulse width modulation (PWM) controller signal of the slider, respectively. Such vibration sources commonly exist in the experimental environment, so noise cancelation is necessary when it comes to precise pressure control or sensitive manipulations.

**Figure 6:**
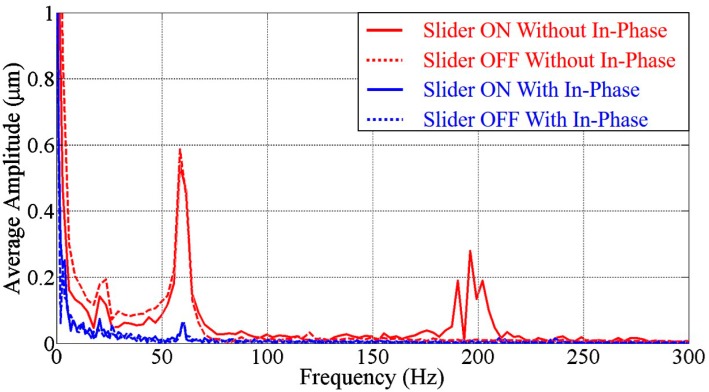
The frequency spectrum without and with the slider motor power turning on.

Figure 6 shows the frequency spectrum by turning the slider power on and off. The colors red and blue show the results without and with the in-phase mechanism, while the solid and dashed lines indicate the conditions with the slider turned off and on, respectively. An interesting observation is that the frequency of 180 Hz disappeared when the power of the slider was turned off. This shows that the noise with 180 Hz is very possibly caused by the slider controller. In addition, the results of the system with the in-phase noise cancelation are not much different whether the slider is on or off. Hence, the results in Figure 4, Figure 5 and Figure 6 support the effectiveness of the in-phase noise cancelation.

### 4.2. The Proposed System for Cell Manipulation

**Figure 7:**
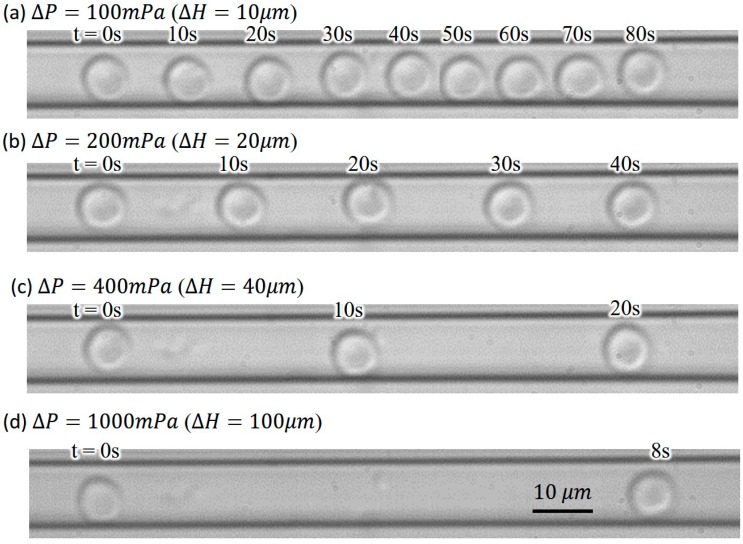
Series of photos of cell position with respect to time under various ΔH: (**a**) ΔH=10 μm; (**b**) ΔH=20 μm; (**c**) ΔH=40 μm; (**d**) ΔH=100 μm.

Figure 7 shows examples of captured image frames from the experiments with RBCs, and Figure 7a–d are driven by the water heads of ΔH = 10, 20, 40 and 100 µm, respectively. Each image in Figure 7 is combined from multiple frames at the times noted on the top of each cell. A greater water head ΔH corresponds to a higher pressure difference ΔP, and results in a faster cell movement. It is because the RBCs are suspended and moved along with the fluid. The RBC velocities can be directly seen from the time and distance between any two RBCs in combined images in Figure 7. For example, the time for the RBCs moving from the most left to the most right in Figure 7a,c are 80 s and 20 s, respectively. If the total travel distances of the two RBCs are assumed to be the same, the velocity of the RBC in Figure 7c is four times faster than the one in Figure 7a.

.

**Figure 8:**
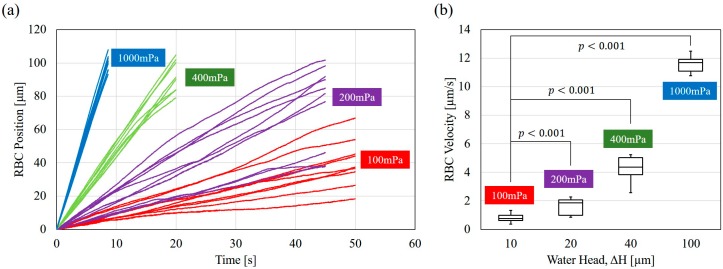
RBC velocity analysis. (**a**) The tracked cell position with respect to time under different ΔH; (**b**) RBC velocity distribution based on tracked results.

Figure 8a shows tracked cell positions on the water head conditions shown in Figure 7. The maximum recording rate of the camera is 250 frames per second (fps) for sufficient temporal resolution. The cell position is automatically tracked using image processing, and the position of zero is set at the position when a cell is first detected. Figure 8a shows that RBC velocities are fairly constant under given pressures ranging from 100 to 1000 mPa, as photos show in Figure 7. The $R^{2}$ values of the linear regression for position-time profiles range from 0.939 to 0.999 with the average value of 0.994. This result shows that the pressure fluctuation is small because the cells move at constant velocities. Figure 8b shows the average RBC velocities from the results in Figure 8a. Student’s *t*-test is applied to the velocities. Significant differences (*p* < 0.1%) between RBC velocities under different water heads are obtained, although velocity variations are observed within each group of Δ*H*. Even a 100 mPa difference has a *p*-value smaller than 0.001. This result supports that pressures under different ΔH can be clearly distinguished. According to Figure 7 and Figure 8, the velocity of RBCs can be controlled by even a few micrometer changes of the water head. The results support the feasibility of the system for precise pressure manipulation.

**Figure 9:**
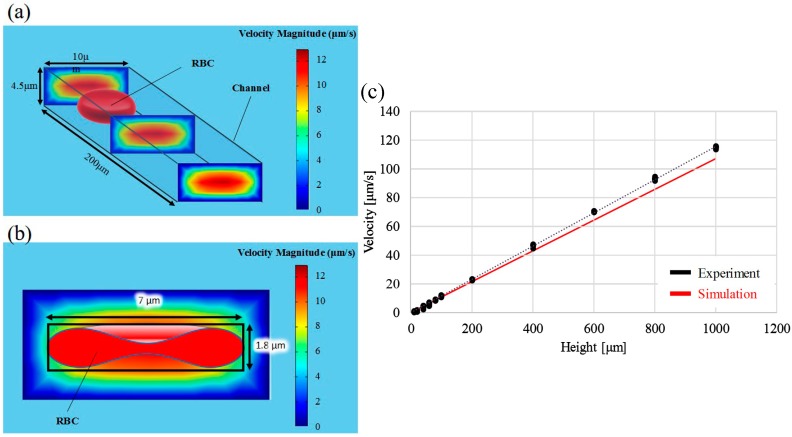
The comparison between theoretical and experimental results: (**a**) The simulated distribution of flow velocity in the microchannel from a 3-dimensional view; (**b**) The theoretical RBC velocity is estimated by the average flow velocity over the occupied area of the RBC in a cross-section; (**c**) Comparisons to the experimental results obtained with the water heads from ΔH=10 μm to ΔH=1000 μm.

Figure 9 shows the flow simulation and the comparison between the results from the experiments and simulation. Flow speed in the microchannel is simulated using commercial software COMSOL Multiphysics (COMSOL Inc., Stockholm, Sweden) for confirming driving pressure in the experiments. Figure 9a shows an example of the velocity profile in a simulated flow with the specified pressure difference ΔP=1 Pa  (corresponding to ΔH=100 μm), and the color on the cross-sections represents the flow velocity as shown in the color bar on the right. The properties of standard saline are used for the simulation, and the width and height of the channel are 10 and 3.5 µm, the same as the ones in the experiments. RBCs are assumed to be suspended at the center of the cross-section with similar geometries, and the RBC velocity in the simulation is calculated as the average velocity over a 7 µm by 1.8 µm area as shown in Figure 9b.

Figure 9c shows the comparison of the RBC velocity between experimental results (dots) and simulated values (line) in the pressure span from 100 mPa to 10 Pa. Although the results are not perfectly matched, they are close and within the same order of magnitude. The comparison evidently supports that the proposed system is capable of manipulating pressure at the mPa level which is greatly beyond the resolution of commercial fluid pressure sensors. According to Figure 9c, the experimental data are slightly greater than the line computed by the simulation. A possible explanation is that the cells are smaller than the area for calculating the average velocity in Figure 9b, so that the simulated velocities are lower than the experimental RBC velocities. We would like to specifically note that the simulation provides only a reference for the order of magnitude in terms of cell velocity. For a real-case simulation, detailed conditions, such as the channel surface, the cell surface, the exact flow resistance along the channel pathway and the shear-induced cell deformation, should be considered.

### 4.3. Preliminary Test on Cell Deformability under Precise Pressure Increments

**Figure 10:**
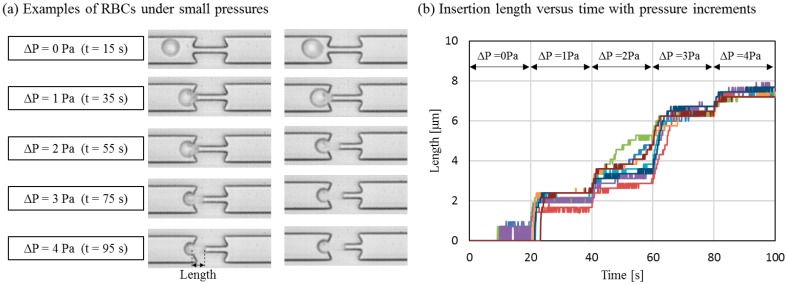
Applications on precise cell evaluation by pressure increments. (**a**) Two examples of RBC deformation under different amounts of applied pressures; (**b**) Tracked insertion length.

Preliminary experiments on the cell deformability test using the proposed system have been carried out. Figure 10a shows two examples of tested RBCs. A 3.5 µm narrow channel is used for providing a geometric constraint in order to deform the RBCs. Each RBC is driven by the applied pressure to squeeze into the narrow channel. The pressure is increased from 0 to 4 Pa with an incremental pressure of 1 Pa. Pressure after each increment is maintained for 20 s, so that each RBC has time to respond to the new applied pressures. The increment of 1 Pa is chosen because RBCs usually start to deform around 1 Pa of pressure, and a pressure less than 1 Pa does not cause much deformation in this setup. The insertion length, as indicated in Figure 10a is measured when the deformation reaches an equilibrium, and is used as an index of cell deformability. 

Figure 10b shows the measured insertion length for eight different RBCs in the deformability test. The spatial resolution of the measurement is 0.24 µm which corresponds to one pixel in the images. It is observed that different amounts of RBC insertions result from the same applied pressure on different RBCs. The difference is especially noticeable at the initial deformation when the pressure is lower than 3 Pa. The insertion length of the RBCs becomes similar when the applied pressure is more than 4 Pa. In addition, this preliminary test also demonstrates the feasibility of applying the proposed system on cell studies.

## 5. Discussion

The clear increase of the insertion length under incremental pressures can be seen in Figure 10. The precise behavior of the cell at the entrance of a narrow channel is very challenging to observe using conventional systems, but is now successfully achieved with the proposed system. The difference between the initial deformation and the large deformation may have different physical meanings in terms of the cytoskeleton and intracellular pressure of a cell. Further experimental studies on RBCs are needed for realizing the governing mechanism of the behavior of how a cell squeezes into a capillary-like channel step by step. 

It is also well known that the PDMS chip is deformable and geometries of a microfluidic channel may increase or decrease under different applied pressures. Channel deformation may affect cell evaluation in conventional approaches, but can be minimized or even neglected if a very low pressure is applied instead. The proposed system can be used as such a low pressure source for other microfluidic applications

## 6. Concluding Remarks

A precise cell manipulation system with in-phase noise cancelation aiming to evaluating cell deformability under pascal-level pressure in a microfluidic system has been developed and experimentally tested. The in-phase noise cancelation is implemented for stabilizing the applied pressure and its effectiveness has been verified using microbeads movement. The system was also applied for cell manipulation and deformability testing using RBCs. The experimental and simulation results show that the proposed system can manipulate pressure at a resolution of 100 mPa, which is a thousand times better than feedback pumps with commercial fluid pressure sensors. We believe that the proposed control system can be practically useful for single-cell manipulation.
